# Beyond the NCCN Risk Factors in Colon Cancer: An Evaluation in a Swedish Population-Based Cohort

**DOI:** 10.1245/s10434-019-08148-3

**Published:** 2020-01-01

**Authors:** Erik Osterman, Artur Mezheyeuski, Tobias Sjöblom, Bengt Glimelius

**Affiliations:** 1 Department of Immunology, Genetics and Pathology, Uppsala University, Uppsala, Sweden; 2 Department of Surgery, Gävle Hospital, Gävle, Sweden; 3 Department of Oncology-Pathology, Karolinska Institute, Stockholm, Sweden; 4 Department of Oncology, Uppsala University Hospital, Uppsala, Sweden

## Abstract

**Background:**

The purpose of this study was to investigate whether pT3–4 and pN-subclassifications, lymph-node ratio (LNR), tumour deposits, pre- and postoperative carcinoembryonic antigen (CEA), and C-reactive protein (CRP)—all parameters commonly collected in clinical management—add information about recurrence risk against a background of routine clinicopathological parameters as defined by the NCCN.

**Methods:**

The prospective cohort consisted of all 416 patients diagnosed with colon cancer stage I–III in Uppsala County between 2010 and 2015. Cox proportional hazard models were used to calculate hazard ratios for time to recurrence and overall survival. The results were compared with the entire Swedish population concerning parameters recorded in the national quality registry, SCRCR, during the same time period.

**Results:**

The Uppsala cohort was representative of the entire Swedish cohort. In unadjusted analyses, pT3-subclassification, pN-subclassification, LNR, tumour deposits, elevated postoperative CEA, and preoperative CRP correlated with recurrence. After adjusting for T-, N-stage, and NCCN risk factors, pN-subclassification, sidedness, and elevated postoperative CEA levels correlated with recurrence. Survival correlated with parameters associated with recurrence, LNR, and elevated postoperative CRP.

**Conclusions:**

Additional information on recurrence risk is available from several routinely recorded parameters, but most of the risk is predicted by the commonly used clinicopathological parameters.

**Electronic supplementary material:**

The online version of this article (10.1245/s10434-019-08148-3) contains supplementary material, which is available to authorized users.

Risk factors of colon cancer recurrence and consequently increased mortality have been identified in multiple studies and incorporated into clinical guidelines^1^,^2^. Most important and universally agreed upon are emergency presentation (particularly perforation but also obstruction), pT4 lesions, the number of metastatic lymph nodes, inadequate number of sampled lymph nodes (< 12), poorly differentiated histology, and vascular and perineural invasion^1^–^3^. Covariation complicates risk assessment in the individual patient, and available factors are considered insufficient to meet present requirements for stratification into risk groups for adjuvant treatment^4^. Other potential prognostic factors, practically always recorded, are side of origin, depth of tumour invasion, lymph-node ratio (LNR), and levels of pre- and postoperative carcinoembryonic antigen (CEA) and C-reactive protein (CRP)^2^,^5^–^20^.

We have recently shown in the entire Swedish population that the recurrence risk after colon cancer surgery today is lower than previously recorded, due to improvements in staging and surgery^21^. Compared with data from the Swedish Colorectal Cancer Registry (SCRCR), which has a high level of coverage but lacks detailed recording of all routinely collected clinicopathological variables, a more complete and comprehensive evaluation can be done by analysing data from a prospective collection in one Swedish county.

## Purpose and Hypotheses

This study was designed to investigate the value of assessing the sidedness, pT3- and pT4-subclassification, LNR, tumour deposits, pre- and postoperative CEA and CRP levels in predicting recurrence risk, and overall survival against a background of routinely recorded clinicopathological variables included in clinical guidelines and nomograms^21^,^22^. The hypothesis was that these emerging risk factors are useful in assessing risk before the initiation of adjuvant chemotherapy in patients with stage II and III colon cancer.

## Methods

### Material

**Fig. 1:**
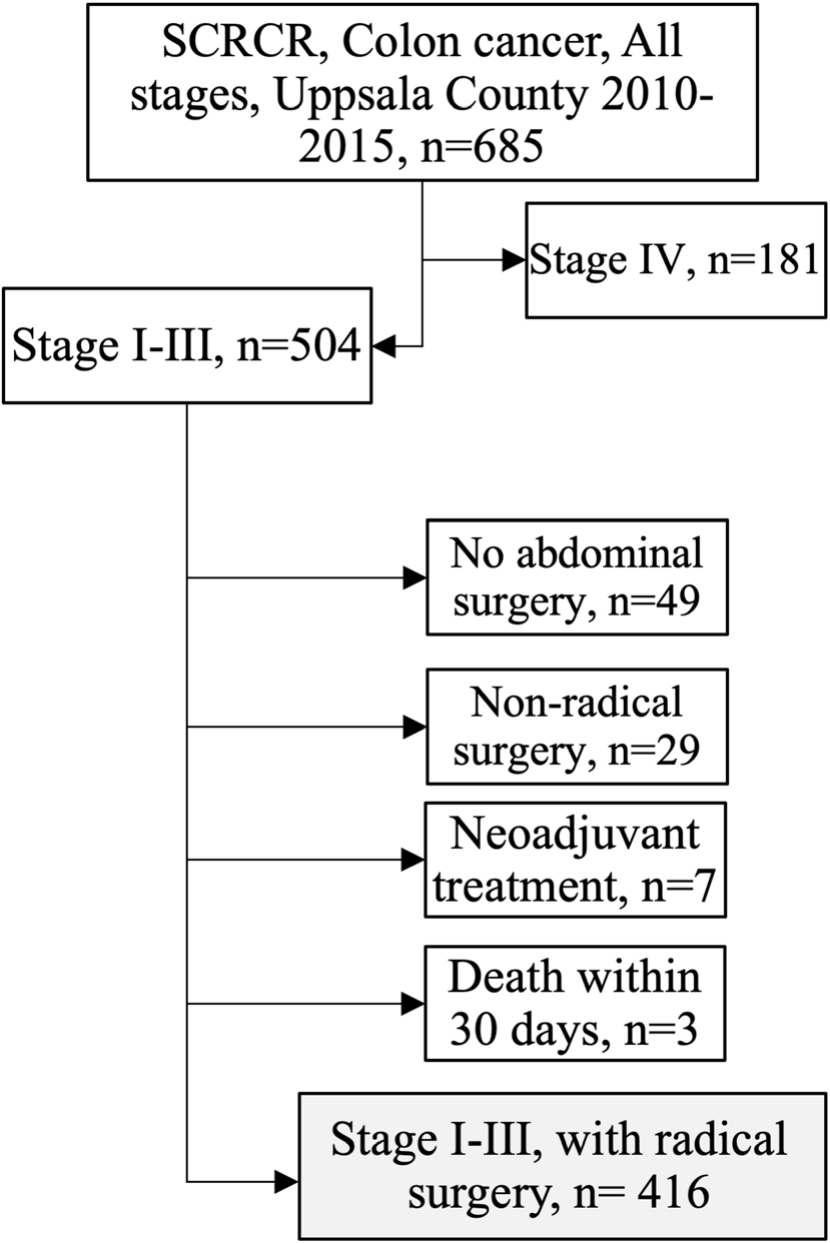
Cohortogram. All patients from Uppsala County who had surgery for colon cancer between 2010 and 2015 were included

The regional ethical review board in Uppsala has approved this study (2010/198, 2014/419, and 2018/490). The study was performed in accordance with the Declaration of Helsinki. The cohort consisted of all 685 patients diagnosed with colon adenocarcinoma (ICD-code C18 and C19) in the Swedish Colorectal Cancer Registry (SCRCR) from Uppsala County (population 349,000 in 2014) that had surgery between January 2010 and December 2015^23^,^24^. Of these, 504 patients had TNM7/UICC stage I–III disease. Eighty-eight patients were excluded: 35 nonresected, 29 with nonradical resection, 14 where polypectomy was performed, 7 who received neoadjuvant chemotherapy because of initially nonresectable disease, and 3 who died within 30 days of surgery. Final cohort size was 416 patients (Fig. 1).

Outcomes and variables were collected from the SCRCR in November 2018 and from electronic patient records and pathology reports; if missing, cases were assessed by a trained gastrointestinal pathologist (AM). Electronic patient records were checked in February 2019 for patients whose surgery was performed in December 2015 to ensure at least 3 years of follow-up. Times were censored at the last data-point for patients where no terminal event had occurred. Predictor variables available in the SCRCR were sidedness, pN-subclassification, and LNR. Not completely recorded in the SCRCR, but gathered from electronic patient records, pathology reports and pathologic reevaluation were pT3- and pT4-subclassification, TDs, CEA, and CRP. Tumours were considered right-sided if proximal to the splenic flexure. pT3-stage was subclassified by depth of invasion into the pericolic tissue (< 1 mm, 1–5 mm, 5–15 mm, and > 15 mm beyond the muscularis propria, a–d)^13^. LNR was calculated from the number of metastatic nodes divided by the number of examined nodes^14^. Dichotomous variables were created for CEA with the cutoff at 5 ng/ml and for CRP with the cutoff at 10 mg/ml^20^. Postoperative blood samples drawn 10 weeks or later were excluded. Baseline variables were age, sex, comorbidities, emergency presentation, ≥ 12 surveyed lymph nodes, grade of malignancy, vascular, and perineural invasion. Comorbidities were available as American Society of Anesthesiologists physical status classification (ASA-PSC)^25^. Outcomes, namely time to recurrence (TTR) and overall survival (OS), were defined as suggested by Punt et al.^26^.

### Statistics

The representability of the smaller county material concerning the baseline parameters recorded in the national SCRCR cohort from 2010 to 2014 consisting of 12,446 radically resected stage I–III patients and their importance for the outcomes, TTR and OS, was tested. The additional value of the more detailed information possible to achieve in a prospective cohort concerning the predictor variables were then tested as detailed below.

The two-sided asymptotic Pearson’s *χ*^2^[2] test was used to test for differences in distribution of outcome, baseline and predictor variables. Fischer’s exact test was used when the Pearson’s *χ*^2^[2] test was inappropriate (cell count ≤ 5). Spearman’s rank correlation coefficient was used to test correlations between continuous variables. The Mann–Whitney *U* test and the Kruskal–Wallis test were used to assess differences in distribution of continuous variables between groups.

Unadjusted and adjusted hazard ratios (HR) for recurrence and mortality were calculated using the Cox proportional hazards model. Emerging predictor variables were tested with adjustment for the baseline model consisting of emergency surgery, pT- and pN-classification, lymph node sampling, malignancy grade, vascular and perineural invasion, and adjuvant treatment (because it decreases recurrence risk and improves survival). Age, sex, and ASA-PSC were added to the model when OS was analysed. Concordance values were calculated for the linear predictor score (Xβ) to assess goodness-of-fit for the adjusted models^27^. Cases with missing values were included in the analyses, with the missing factor set to missing. For subgroup analyses, risk factors described by the National Comprehensive Cancer Network (NCCN) (emergency surgery, including obstruction and perforation, low lymph node yield, high-grade malignancy, and vascular and perineural invasion) were used to define three risk groups of particular interest: (1) a truly low-risk group, pT3N0 with no risk factor, where adjuvant treatment is not recommended and seldom administered; (2) a low-risk group, pT3N0 with 1 risk factor; and (3) an intermediate-risk group, pT4N0 and pT1–3N1 with no other risk factor, where the use of adjuvant treatment is recommended in guidelines but could be discussed due to low recurrence risks according to the recent Swedish national study described above 21.

Statistics were analysed with IBM SPSS Statistics for Macintosh, Version 25.0 (Armonk, NY: IBM Corp). Results were considered statistically significant if *p* < 0.05.

## Results

### Cohort Characteristics and Representativity of the County Cohort

There was an equal distribution between male and female patients in the cohort, and the mean age was 72 years (SD = 12). Median follow-up for patients who did not have an event was 5.5 years (minimum 3 years). Fifteen percent (63/416) were in stage I, 39% (163/416) in stage II, and 46% (190/416) in stage III. Thirty-six percent (148/416) of the patients received adjuvant chemotherapy: no patients in stage I, 15% in stage II (25/163), and 65% (123/190) in stage III. Oxaliplatin was administered in 54% of cases (80/148) treated with adjuvant chemotherapy.

Compared with the national cohort, mean age, sex distribution, and tumour side did not differ (Supplementary Table 1). However, there were slightly more T1–2 and N0 tumours (stage I) in the national material. Missingness for these variables was low in both materials. Malignancy grade, vascular, and perineural invasion were missing in a higher proportion in the national material (6–12%) compared with the county material (3–5%), and the percentage of complete cases regarding the above basic variables was 83% in the national and 95% in the county material. Univariable hazards for TTR and OS of the above variables are presented in Supplementary Table 1, which shows that the risks were very similar between the national cohort and the county cohort.

### Recurrence and Mortality in the County Cohort

Distribution of recurrences and mortality for the predictor variables are presented in Table 1. Emergency presentation, elevated preoperative CRP, advanced pT- and pN-stage, high-grade malignancy, vascular and perineural invasion, and elevated postoperative CEA were statistically significantly associated with recurrence. Age older than 70 years, emergency surgery, high ASA-PSC, pT- and pN-stage, vascular and perineural invasion, pre- and postoperative CEA > 5 ng/ml, and postoperative CRP > 10 mg/l were associated with mortality.

**Table 1:** Patient characteristics of the cohort

Parameter	Total		Recurrence			Mortality		
	No.	(%)	No.	(%)	*p*	No.	(%)	*p*
Total	416	(100)	79	(19)		135	(32)	
Age (year)								
< 70	173	(42)	31	(18)	0.638	32	(18)	< 0.001
> 70	243	(58)	48	(20)		103	(42)	
Sex								
Male	208	(50)	37	(18)	0.532	71	(34)	0.464
Female	208	(50)	42	(20)		64	(31)	
ASA								
1	72	(17)	11	(15)	0.653	12	(17)	< 0.001
2	187	(45)	36	(19)		50	(27)	
3–4	157	(38)	32	(20)		73	(46)	
Surgery								
Elective	341	(82)	51	(15)	< 0.001	99	(29)	0.001
Emergency	75	(18)	28	(37)		36	(48)	
Sidedness								
Right	230	(55)	50	(22)	0.112	81	(35)	0.180
Left	186	(45)	29	(16)		54	(29)	
pT								
pT1	35	(8)	0	(0)	0.001 F	6	(17)	< 0.001F
pT2	37	(9)	4	(11)		11	(30)	
pT3a	49	(12)	7	(14)		11	(22)	
pT3b	102	(25)	16	(16)		27	(26)	
pT3c	77	(19)	16	(21)		21	(27)	
pT3d	39	(9)	12	(31)		19	(49)	
pT3NA	1	(0)	0	(0)		1	(100)	
T4a	49	(12)	17	(35)		26	(53)	
T4b	27	(6)	7	(26)		13	(48)	
pN								
pN0	226	(54)	20	(9)	< 0.001	56	(25)	0.001
pN1a	53	(13)	13	(25)		21	(40)	
pN1b	56	(13)	12	(21)		20	(36)	
pN1c	14	(3)	2	(14)		4	(29)	
pN2a	34	(8)	16	(47)		14	(41)	
pN2b	33	(8)	16	(48)		20	(61)	
Sampled nodes								
< 12	38	(9)	3	(8)	0.067	10	(26)	0.397
≥ 12	378	(91)	76	(20)		125	(33)	
TD								
No	337	(81)	57	(17)	0.056	103	(31)	0.233
Yes	56	(13)	17	(30)		23	(41)	
Malignancy grade								
Low	350	(84)	59	(17)	0.011	107	(31)	0.059
High	66	(16)	20	(30)		28	(42)	
Mucinous								
No	338	(81)	66	(20)	0.158	108	(32)	0.691
Yes	63	(15)	13	(21)		23	(37)	
Vascular invasion								
No	291	(70)	35	(12)	< 0.001	82	(28)	0.005
Yes	112	(27)	41	(37)		45	(40)	
Perineural invasion								
No	343	(82)	51	(15)	< 0.001	98	(29)	0.001
Yes	52	(13)	23	(44)		26	(50)	
*CEA (ng/ml)*								
Preoperative								
< 5	195	(47)	30	(15)	0.207	49	(25)	0.010
> 5	110	(26)	25	(23)		41	(37)	
Postoperative								
< 5	220	(53)	36	(16)	0.001	59	(27)	0.021
> 5	23	(6)	11	(48)		11	(48)	
CRP (mg/l)								
Preoperative								
< 10	211	(51)	35	(17)	0.002	60	(28)	0.084
> 10	136	(33)	38	(28)		54	(40)	
Postoperative								
< 10	204	(49)	36	(18)	0.505	59	(29)	< 0.001
> 10	48	(12)	12	(25)		28	(58)	
Adjuvant chemotherapy								
No	268	(64)	35	(13)	< 0.001	96	(36)	0.048
Yes	148	(36)	44	(30)		39	(26)	

Number of cases for each category of the variable and percent of cases in each category. (# by cohort size). Outcomes recurrence and mortality reported with number of cases and the percent within each category. Cases with missing data not reported. *P* values are calculated with Pearson’s *χ*^2^ test or the Fisher’s exact test (*F*)

Adjusted proportional hazards regression of the baseline model for TTR (emergency surgery, pT- and pN-classification, lymph node sampling, malignancy grade, vascular and perineural invasion, adjuvant treatment) and OS (TTR-baseline model and age, sex and ASA-PSC) are presented in Table 2. Initiation of adjuvant therapy was associated with more recurrences but fewer deaths. Adjuvant therapy correlated with better OS but not significantly with recurrence risk in the baseline model.

**Table 2:** Baseline model for TTR and OS

Factor	Level	TTR				OS			
		HR	CI-	CI+	*p*	HR	CI−	CI+	*p*
Age	Continuous					1.0	1.0	1.0	0.025
Sex	Male					(Ref)			
	Female					0.9	0.7	1.3	0.739
ASA	1					(Ref)			0.004
	2					1.3	0.6	2.5	0.493
	3–4					2.3	1.2	4.7	0.015
Surgery	Elective	(Ref)				(Ref)			
	Emergency	2.5	1.5	4.1	< 0.001	1.5	1.0	2.3	0.061
pT	pT1–2	(Ref)			0.578	(Ref)			0.001
	pT3	1.7	0.6	5.0	0.368	0.9	0.5	1.7	0.853
	pT4	1.9	0.6	6.1	0.296	2.1	1.1	4.1	0.03
pN	pN0	(Ref)			< 0.001	(Ref)			< 0.001
	pN1	2.4	1.2	4.6	0.011	2.2	1.4	3.4	0.001
	pN2	5.8	2.8	12.1	< 0.001	4.2	2.4	7.5	< 0.001
Sampled nodes	< 12	(Ref)				(Ref)			
	≥12	2.0	0.6	6.8	0.241	1.6	0.8	3.2	0.158
Malignancy grade	Low	(Ref)				(Ref)			
	High	1.6	0.9	2.8	0.078	0.9	0.6	1.5	0.787
Vascular invasion	No	(Ref)				(Ref)			
	Yes	1.4	0.8	2.5	0.199	0.9	0.6	1.5	0.721
Perineural invasion	No	(Ref)				(Ref)			
	Yes	1.7	1.0	3.0	0.056	1.6	1.0	2.7	0.072
Adjuvant treatment	No	(Ref)				(Ref)			
	Yes	0.6	0.4	1.1	0.105	0.4	0.2	0.7	0.001

Multivariable model for TTR and OS used in adjusted analyses of predictor variables

### Emerging Risk Factors

Emerging risk factors are presented in Table 3 with the associated hazard ratios, 95% confidence intervals, and statistical significance from unadjusted and adjusted (with baseline model) analyses. Concordance value for the baseline models were 0.77 for TTR and 0.79 for OS. No statistically significant differences were seen when the emerging variables were added one by one (Supplementary Table 2).

**Table 3:** Cox proportional hazards regression for TTR, for predictor variables

TTR		Unadjusted				Adjusted			
Factor	Level	HR	CI-	CI+	*p*	HR	CI-	CI+	*p*
Side	Right	(Ref)				(Ref)			
	Left	0.7	0.4	1.1	0.088	0.5	0.3	0.9	0.016
pT	pT1–2	(Ref)			0.002	(Ref)			0.898
pT3	a	2.7	0.8	9.2	0.115	1.6	0.4	5.9	0.469
	b	2.9	1.0	8.8	0.053	1.6	0.5	5.0	0.454
	c	4.1	1.4	12.4	0.011	1.5	0.4	4.8	0.534
	d	6.9	2.2	21.5	0.001	2.3	0.7	8.1	0.181
pT4	a	8.0	2.7	23.9	< 0.001	2.1	0.6	7.2	0.235
	b	6.3	1.8	21.4	0.003	1.9	0.5	7.0	0.357
pN	0	(Ref)			< 0.001	(Ref)			< 0.001
	1a	3.4	1.7	6.8	0.001	2.6	1.2	5.7	0.013
	1b	2.8	1.4	5.7	0.005	2.3	1.0	5.1	0.044
	1c	1.7	0.4	7.3	0.470	1.5	0.3	6.9	0.600
	2a	6.5	3.4	12.5	< 0.001	5.4	2.4	12.0	< 0.001
	2b	9.3	4.8	18.0	< 0.001	6.4	2.8	14.9	< 0.001
LNR	0–1	37.2	15.6	88.6	< 0.001	3.6	0.7	18.0	0.119
TD	No	(Ref)				(Ref)			
	Yes	2.2	1.3	3.8	0.004	0.8	0.4	1.5	0.437
CEA	ng/ml								
Preoperative	< 5	(Ref)				(Ref)			
	> 5	1.7	1.0	2.8	0.063	1.2	0.7	2.2	0.464
Postoperative	< 5	(Ref)				(Ref)			
	> 5	3.9	2.0	7.6	< 0.001	2.6	1.2	5.6	0.018
CRP	mg/l								
Preoperative	< 10	(Ref)				(Ref)			
	> 10	1.9	1.2	3.0	0.008	1.0	0.6	1.7	0.994
Postoperative	< 10	(Ref)				(Ref)			
	> 10	1.8	1.0	3.5	0.067	1.8	0.9	3.9	0.108

Variables tested one by one (unadjusted), and with the baseline model (adjusted)

*HR* hazard ratio; *CI* confidence interval, with lower and upper bounds reported; *LNR* lymph node ratio (positive nodes by found nodes)

#### Sidedness

Left-sided tumours were associated with young age, male sex, emergency surgery, fewer than 12 lymph nodes investigated, and low-grade malignancy. No correlation with recurrence was seen in unadjusted analyses, but when adjusting for baseline factors, HR for recurrence in left-sided tumours was significantly lower. In unadjusted analyses by stage, sidedness was not correlated to recurrences. When adjusting for the baseline model, left-sided tumours correlated with lower risk of recurrence in stage II [hazard ratio (HR) 0.18, 95% confidence interval (CI) 0.04–0.79] but not in stage I or III.

#### pT Stage and Substages

Advanced pT-stage was associated with emergency surgery, right-sided tumour, advanced pN-stage, more than 12 nodes investigated, high-grade malignancy, vascular and perineural invasion, and administration of adjuvant treatment. In unadjusted analyses, HR increased for every increase in pT3-substage but not between pT4a and pT4b.

#### Lymph Nodes

Higher LNR and more advanced pN-stage was associated with emergency surgery, increasing pT-stage, high-grade malignancy, vascular and perineural invasion, and elevated preoperative CEA and CRP. Optimal cutoff for LNR to stratify recurrences as calculated by receiver operating curve analysis was 0.13 (Supplementary Figure 1, AUC 0.65, 95% CI 0.56–0.74); 44% (83/190) of stage III patients were then categorised as LNR-high. Of these, 46% (39/83) had a recurrence in contrast to 19% (20/107) of LNR-low patients. LNR correlated strongly with recurrence in unadjusted (HR 37.2) but not in a baseline model adjusted analysis. All node-positive subclassifications of the pN-stage, except for pN1c (presence of TD, but otherwise pN0), correlated with recurrence in both unadjusted and adjusted analyses. No differences could be seen between pN1a and b.

#### Tumour Deposits

The presence of TDs was associated with emergency surgery, left-sided cancer, high pT- and pN-stage, and later administration of adjuvant treatment. TDs correlated with recurrence in unadjusted but not in baseline model adjusted analyses. No difference in recurrence rates were seen when TDs were stratified by lymph-node involvement.

#### CEA

Mean preoperative CEA was 16 ng/ml (SD = 71). The median was 3 ng/ml (range 0.3–995). Mean postoperative CEA was 3 ng/ml (SD = 7). The median was 2 ng/ml (range 0–98). Distribution of recurrences is presented in Table 1.

Elevated preoperative CEA was associated with old age, female sex, high ASA-PSC, advanced pT- and pN-stage and vascular invasion. Preoperative CEA did not correlate with recurrence. Elevated postoperative CEA was associated with vascular invasion and correlated with recurrences in unadjusted and baseline model adjusted analyses.

#### CRP

Mean preoperative CRP was 21 mg/l (SD = 39). The median was 6 mg/l (range 0.3–241). Mean postoperative CRP was 10 mg/l (SD = 24). The median was 2 mg/l (range 0.2–232). Distribution of recurrences is presented in Table 1. Elevated preoperative CRP was associated with old age, female sex, high ASA-PSC, advanced pT- and pN-stage, high-grade malignancy, and vascular invasion. Elevated postoperative CRP was associated with high ASA-PSC, emergency surgery, and postoperative complications. Preoperative CRP correlated with recurrence in unadjusted but not in baseline model adjusted analysis. Postoperative CRP did not correlate with recurrence.

### Risk Groups

Fifty-seven percent of stage II patients (93/163) were identified as truly low-risk (pT3N0 with no NCCN risk factor) of which 10% (9/93) recurred. Chemotherapy was initiated in five patients; three recurred. Twenty-four percent of stage II patients were in the low-risk group, pT3N0 with one risk factor (29/163), chemotherapy was initiated in seven patients, and no patients recurred (0/29). In the intermediate-risk group, ten patients were pT4N0 without risk factors and 40 patients pT1–3N1 without risk factors; overall 20% of these patients recurred (10/50). Chemotherapy was initiated in 27 patients, and 11% (3/27) recurred.

There were no statistically significant differences in distribution of sidedness, pT3-subclassification, CEA, and CRP between patients with and without recurrence within these three risk groups. There was a strong covariation of risk factors among the pT3N0 patients; 79% (70/89) with pT3a-b disease were classified as truly low-risk, whereas 49% (17/47) of the pT3c–d patients were classified as truly low-risk. Overall, 49% of pT3a (24/49) and 45% of pT3b (46/102) were N0 and truly low-risk, whereas only 23% of pT3c (18/77) and 13% of pT3d patients (5/39) were N0 and truly low-risk.

### Overall Survival

Age, high ASA-PSC, right-sided tumours, pT3d-, pN1a-, and pN2b-subclassification, increased LNR, high-grade malignancy, vascular and perineural invasion, no adjuvant treatment, pre- and postoperative CEA, and CRP correlated with increased hazard of mortality in unadjusted analyses. Right-sided tumours, pT4, node positivity (except pN1c), LNR, postoperative CEA, and CRP correlated with increased hazard of mortality when adjusting for baseline variables. Results from the adjusted analysis are presented in Supplementary Table 3, and the baseline model is presented in Table 2.

## Discussion

Improvements in the care of colon cancer patients, subsequent improvements in prognosis and the wish for personalized medicine require better risk stratification before initiation of adjuvant therapy^3^. Against the baseline of clinicopathological variables recommended in guidelines (like NCCN and ESMO), there is a potential value in assessing sidedness, pT3-subclassification, pN-subclassification, and postoperative CEA when discussing the value of administrating adjuvant chemotherapy and its duration in radically operated colon cancer patients^1^,^2^. All emerging factors were associated with worse clinical features; however, only some correlated with increased recurrence risk in adjusted analysis, meaning that they are rather signs of advanced disease and not independent features. The association between factors is evident in the risk groups where the value of adjuvant chemotherapy could be discussed; more pT3a–b were classified as truly low-risk than pT3c–d and the recurrence risks were not different between pT3-substages within these risk groups. When analysing the whole cohort, high pT-stage and substages were associated with several negative prognostic factors, but when adjusting for these, pT-stage was nonsignificant for recurrence. pT3d seems as bad as pT4a and b substage regarding distribution of both recurrences and mortality, telling that the important distinction is not between pT3 and pT4 but within the pT3-tumours. pT3a and b were associated with low risk, node-negative disease, whereas pT3c and definitely pT3d disease was associated with high-risk features and node involvement. Peritoneal involvement (pT4a) correlated with worse HR compared with involvement of other structures (pT4b) confirming what was seen in the national material and another recent large patient series^21^,^28^.

Elevated preoperative CEA indicated more advanced disease, as described previously and included in previous NCCN guidelines, but in this material of patients after radical surgery there was no difference in recurrence rates^1^,^16^,^29^. In this study, elevated CEA within 6–10 weeks after surgery correlated with recurrence risk, suggesting that it indicates residual disease. Like preoperative CEA, CRP indicated advanced disease but was not an inherent risk factor. It has recently been recognized that the side of the primary tumour is important; however, controversy exists on which side is worse^5^,^7^,^9^. We found that left-sided cancers had fewer recurrences when adjusting for baseline factors, including node positivity. When analysed by stage, it correlated in adjusted analysis in stage II but not in stage III. The equivocal results in literature, and the somewhat inconsistent results seen here, tell that sidedness cannot be a strong factor for predicting recurrence and thus the need for adjuvant therapy. LNR and TDs had a strong correlation with recurrence in unadjusted analyses; however, both were nonsignificant in adjusted analysis. Factors associated with recurrence were also correlated with poor survival (sidedness, pT and pN, elevated postoperative CRP). Potentially as a reflection of the host immune response, there was a correlation between elevated preoperative CRP and increased survival^30^. Initiation of adjuvant treatment correlated with better overall survival but not decreased recurrence risk, indicating that therapy was given selectively to high-risk patients. However, the goal was not to investigate treatment effects, instead it was treated as a confounder throughout analyses.

There was no missing data regarding OS and TTR, and median follow-up in event-free patients was 5.5 years (minimum 3 years), within which time most recurrences have happened in both stage II and III disease according to larger studies^31^. The material was comparably small, influencing both the total number of events (79 recurrences, 139 deaths) per factor (12) and the overall statistical power but truly population-based. To account for the small material, we compared it with the entire national material and found a similar distribution of age, gender, ASA-PSC, stage, and emergency presentation, indicating representability. The lower proportion of T1–2 and N0 patients could be explained by the inclusion of patients who had a polypectomy in the national material. More patients had 12 or more lymph nodes evaluated in the present study than in the national material (91% vs. 88%), which might be an effect of a more thoroughly staged cohort. Increased lymph node harvests was seen in the national material covering 2010–2014, used here, versus 2007–2012, reported previously^21^. The county data was more detailed and missingness lower, which improve the validity. In addition, the baseline model is similar to the model developed in the national material, e.g., the unadjusted HR associated with vascular invasion was 3.0 in the national material and 3.7 in the county material. Some confounding factors have not been measured and adjusted for, because there was no recorded data regarding them, e.g., smoking is known to effect CEA levels. CEA and CRP were available for approximately 80% of patients before surgery and 60% after surgery, with the missing data introducing some uncertainty. The ASA-PSC measures perioperative risk and does not include all comorbidities that might influence survival; however, it does include most systemic diseases. There are risk factors not accounted for; among the five patients with truly low-risk pT3N0 disease given chemotherapy, three recurred—indicating that these patients may have had risk factors known to the clinicians but not completely reported for the purposes of this study.

Further investigations are needed regarding some of these emerging factors, whereas others could make the leap into clinical practice. Controversy still surrounds sidedness. It is known to be of importance in the metastatic setting, but evidence is still conflicting in the early stages. The pT-subclassification is utilised in rectal cancer, but its place in the management of colon cancer patients is still unclear. The cutoff between low and high risk of recurrence seems to be within pT3, whereas the terminology for pT4 subclassification makes little sense if the finding of better outcomes for patients in pT4b than in pT4a holds. Both LNR and TDs (including pN1c) add little when accounting for other factors, especially the already accepted pN-subclassification. CEA is already included in some nomograms and guidelines but primarily as a marker before surgery, indicating advanced disease^2^,^29^. The goal of adjuvant treatment is to eliminate any (microscopic) residual disease and elevated postoperative CEA indicates this. Measuring it before adjuvant treatment not only establishes a baseline but also indicates the likelihood to relapse. This information could be valuable when choosing between a fluoropyrimidine alone or with oxaliplatin, as well as the length of oxaliplatin-containing chemotherapy.

## Conclusions

All of the investigated emerging factors are associated with worse clinicopathological features. pN-subclassification and postoperative CEA independently correlate with the recurrence risk. Assessing these before initiation of adjuvant treatment may improve prognostication, but it is difficult to improve the prediction of recurrence risk beyond what is already routinely recorded and known. Adding genetic and molecular risk factors may improve prognostication and large, prospective, and high-quality biobank projects will hopefully be able to find, and validate, new markers of importance.

## Electronic supplementary material

Below is the link to the electronic supplementary material.

Supplementary material 1 (PDF 75 kb)

Supplementary material 2 (PDF 58 kb)

Supplementary material 3 (PDF 58 kb)

Supplementary material 4 (TIFF 1249 kb)
