# Supplementary material for: Beyond the NCCN Risk Factors in Colon Cancer: An Evaluation in a Swedish Population-Based Cohort
Source: Ann Surg Oncol. 2020 Jan 1;27(4):1036–45. doi: 10.1245/s10434-019-08148-3 (PMC7060230; doi:10.1245/s10434-019-08148-3)
Supplement: Supplementary file 1 — Supplementary material 1 (PDF 75 kb) [file 10434_2019_8148_MOESM1_ESM.pdf]

Supplementary Table 1: Comparison of county cohort and national cohort

**Parameter**		**County 2010-2015**						**SCRCR 2010-2014**					
				**Unadjusted**						**Unadjusted**			
		**No.**	**(%)**	**HR TTR**	**p**	**HR OS**	**p**	**No.**	**(%)**	**HR TTR**	**p**	**HR OS**	**p**
**Total**		416						12446					
**Age (continuous)**	**mean, sd**	72, 12						72, 11					
	**Range**	26-96		1.0	0.964	1.1	<0.001	17-101		1.0	0.587	1.1	<0.001
**Sex**	**Male**	208	(50)	(Ref)		(Ref)		6084	(49)	(Ref)		(Ref)	
	**Female**	208	(50)	1.1	0.541	0.9	0.464	6362	(51)	0.9	0.001	0.9	<0.001
**ASA**	**1**	72	(17)	(Ref)		(Ref)		1636	(13)	(Ref)		(Ref)	
	**2**	187	(45)	1.3	0.470	1.9	0.055	6510	(52)	1.0	0.747	1.8	<0.001
	**3–4**	157	(38)	1.5	0.220	4.0	<0.001	3976	(32)	1.1	0.125	4.3	<0.001
**Surgery**	**Elective**	341	(82)	(Ref)		(Ref)		10438	(84)	(Ref)		(Ref)	
	**Emergency**	75	(18)	3.0	<0.001	1.8	0.004	1843	(15)	2.3	<0.001	2.0	<0.001
**Complications**	**No**	298	(72)	(Ref)		(Ref)		10171	(82)	(Ref)		(Ref)	
	**Yes**	118	(28)	1.5	0.095	1.8	0.002	2275	(18)	1.1	0.039	1.5	<0.001
**pT**	**1–2**	72	(17)	(Ref)	<0.001	(Ref)		2770	(22)	(Ref T1)		(Ref)	
	**3**	269	(65)	3.8	0.010	1.2	0.429	7541	(61)	2.8	<0.001	1.4	<0.001
	**4**	75	(18)	7.2	<0.001	2.9	<0.001	2130	(17)	7.1	<0.001	2.8	<0.001
	**NA**	0	(0)					6	(0)				
**pN**	**0**	226	(54)	(Ref)	<0.001	(Ref)		7845	(63)	(Ref)		(Ref)	
	**1**	123	(30)	2.9	<0.001	1.6	<0.001	2864	(23)	2.8	<0.001	1.5	<0.001
	**2**	67	(16)	7.6	<0.001	2.4	<0.001	1737	(14)	6.7	<0.001	2.6	<0.001
	**NA**	0	(0)										
**Sampled nodes**	**<12**	38	(9)	(Ref)		(Ref)		1243	(10)				
	**≥12**	378	(91)	2.7	0.095	1.4	0.298	10937	(88)	1.1	0.451	0.8	<0.001
**Malignancy grade**	***Low***	350	(84)	(Ref)		(Ref)		9202	(74)	(Ref)		(Ref)	
	High	66	(16)	2.1	0.003	1.6	0.039	2554	(21)	1.5	<0.001	1.5	<0.001
	**Missing**	0	(0)					696	(6)				
**Vascular invasion**	***No***	291	(70)	(Ref)	<0.001	(Ref)		8986	(72)	(Ref)		(Ref)	
	Yes	112	(27)	3.7	<0.001	1.7	0.005	2907	(23)	3.0	<0.001	1.9	<0.001
	Missing	13	(3)					794	(6)				
**Perineural invasion**	***No***	343	(82)	(Ref)	<0.001	(Ref)		9733	(78)	(Ref)		(Ref)	
	Yes	52	(13)	3.6	<0.001	2.0	0.001	1443	(12)	3.3	<0.001	2.1	<0.001
	Missing	21	(5)					1511	(12)				
**Adjuvant treatment**	**No**	268	(64)	(Ref)		(Ref)		8930	(72)	(Ref)		(Ref)	
	**Yes**	148	(36)	2.3	<0.001	0.7	0.027	3516	(28)	2.2	<0.001	0.7	<0.001
**Recurrences**		79	(19)					1965	(16)				
**Mortality**		135	(32)					4010	(32)				
**Complete cases**		394	(95)					10309	(83)				

Subtitle supplementary table 1: Complete cases for the variables described in table 1. Unadjusted HR and p-values for the variables in the baseline models for TTR and OS.
