# Supplementary material for: Beyond the NCCN Risk Factors in Colon Cancer: An Evaluation in a Swedish Population-Based Cohort
Source: Ann Surg Oncol. 2020 Jan 1;27(4):1036–45. doi: 10.1245/s10434-019-08148-3 (PMC7060230; doi:10.1245/s10434-019-08148-3)
Supplement: Supplementary file 2 — Supplementary material 2 (PDF 58 kb) [file 10434_2019_8148_MOESM2_ESM.pdf]

**Supplementary table 2:** Concordance values for the linear predictor score and 95% confidence intervals

| | | TTR | 95% CI | | OS | 95% CI | |
|---|---|---|---|---|---|---|---|
| *Model* | | *Concordance* | *Lower* | *Upper* | *Concordance* | *Lower* | *Upper* |
| | **Baseline (BL)** | 0.77 | 0.71 | 0.83 | 0.79 | 0.74 | 0.83 |
| **BL +** | **Side** | 0.79 | 0.73 | 0.84 | 0.79 | 0.74 | 0.83 |
| **BL +** | **pT substages** | 0.77 | 0.71 | 0.83 | 0.80 | 0.75 | 0.84 |
| **BL +** | **pN substages** | 0.77 | 0.71 | 0.83 | 0.79 | 0.74 | 0.84 |
| **BL +** | **LNR** | 0.77 | 0.71 | 0.83 | 0.79 | 0.74 | 0.83 |
| **BL +** | **TD** | 0.77 | 0.71 | 0.83 | 0.79 | 0.75 | 0.84 |
| **BL +** | **Preoperative CEA** | 0.77 | 0.71 | 0.83 | 0.79 | 0.74 | 0.84 |
| **BL +** | **Postoperative CEA** | 0.78 | 0.72 | 0.84 | 0.79 | 0.74 | 0.83 |
| **BL +** | **Preoperative CRP** | 0.78 | 0.72 | 0.84 | 0.79 | 0.74 | 0.83 |
| **BL +** | **Postoperative CRP** | 0.77 | 0.71 | 0.83 | 0.79 | 0.75 | 0.84 |

Subtitle supplementary table 2: All, except baseline, adjusted for baseline
