# Supplementary material for: Beyond the NCCN Risk Factors in Colon Cancer: An Evaluation in a Swedish Population-Based Cohort
Source: Ann Surg Oncol. 2020 Jan 1;27(4):1036–45. doi: 10.1245/s10434-019-08148-3 (PMC7060230; doi:10.1245/s10434-019-08148-3)
Supplement: Supplementary file 3 — Supplementary material 3 (PDF 58 kb) [file 10434_2019_8148_MOESM3_ESM.pdf]

Supplementary table 3: Cox proportional hazards regression, OS, for investigated variables

OS		Unadjusted				Adjusted			
**Factor**	**Level**	**HR**	**CI-**	**CI+**	**P**	**HR**	**CI-**	**CI+**	**P**
**Side**	**Right**	(Ref)				(Ref)			
	**Left**	0.7	0.5	1.0	0.049	0.6	0.4	0.9	0.024
**pT**	**pT1-2**	(Ref)			<0.001	(Ref)			<0.001
**pT3**	**a**	0.9	0.4	1.9	0.763	0.8	0.4	1.8	0.570
	**b**	1.1	0.6	2.1	0.709	1.0	0.5	1.8	0.887
	**c**	1.1	0.6	2.0	0.864	0.8	0.4	1.6	0.512
	**d**	2.3	1.2	4.4	0.015	1.4	0.6	3.0	0.401
**pT4**	**a**	2.9	1.6	5.4	0.001	2.5	1.2	5.1	0.017
	**b**	2.9	1.4	6.0	0.004	2.0	0.9	4.5	0.085
**pN**	**0**	(Ref)			<0.001	(Ref)			<0.001
	**1a**	1.8	1.1	3.0	0.017	2.2	1.2	3.8	0.007
	**1b**	1.6	1.0	2.7	0.058	2.6	1.5	4.7	0.001
	**1c**	1.0	0.4	2.7	0.964	1.4	0.4	4.2	0.590
	**2a**	1.7	0.9	3.1	0.077	3.0	1.5	6.0	0.002
	**2b**	3.5	2.1	5.9	<0.001	6.5	3.4	12.5	<0.001
**LNR**	**0–1**	8.6	3.8	19.6	<0.001	6.3	1.5	26.2	0.011
**CEA**	**ng/ml**								
**Preoperative**	**<5**	(Ref)				(Ref)			
	**>5**	1.6	1.1	2.5	0.020	1.2	0.8	1.9	0.417
**Postoperative**	**<5**	(Ref)				(Ref)			
	**>5**	2.5	1.3	4.7	0.006	2.3	1.1	4.9	0.033
**CRP**	**mg/l**								
**Preoperative**	**<10**	(Ref)				(Ref)			
	**>10**	1.6	1.1	2.2	0.019	0.8	0.5	1.3	0.357
**Postoperative**	**<10**	(Ref)				(Ref)			
	**>10**	2.8	1.8	4.5	<0.001	2.2	1.3	3.6	0.004
**TD**	**No**	(Ref)				(Ref)			
	**Yes**	1.5	0.9	2.3	0.086	0.8	0.4	1.4	0.375

Subtitle supplementary table 3: Variables tested one by one (unadjusted) or with the baseline model (Adjusted)

LNR: Lymph node ratio (positive nodes by found nodes)

HR: Hazard ratio, CI: 95% Confidence interval, with lower and upper bounds reported.
