# Supplementary figures and images for: Beyond the NCCN Risk Factors in Colon Cancer: An Evaluation in a Swedish Population-Based Cohort
Source: Ann Surg Oncol. 2020 Jan 1;27(4):1036–45. doi: 10.1245/s10434-019-08148-3 (PMC7060230; doi:10.1245/s10434-019-08148-3)

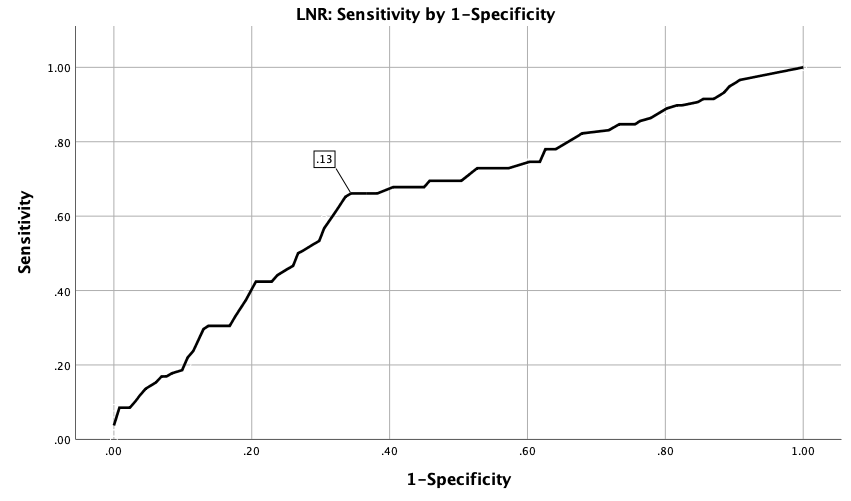

Supplement: Supplementary file 4 — Supplementary material 4 (TIFF 1249 kb) [file 10434_2019_8148_MOESM4_ESM.tiff]
